# Chitosan-Coated Nanoparticles: Effect of Chitosan Molecular Weight on Nasal Transmucosal Delivery

**DOI:** 10.3390/pharmaceutics11020086

**Published:** 2019-02-18

**Authors:** Franciele Aline Bruinsmann, Stefania Pigana, Tanira Aguirre, Gabriele Dadalt Souto, Gabriela Garrastazu Pereira, Annalisa Bianchera, Laura Tiozzo Fasiolo, Gaia Colombo, Magno Marques, Adriana Raffin Pohlmann, Silvia Stanisçuaski Guterres, Fabio Sonvico

**Affiliations:** 1 Programa de Pós-Graduação em Ciências Farmacêuticas, Universidade Federal do Rio Grande do Sul, Porto Alegre 90610-000, Brazil; fbruinsmann@gmail.com (F.A.B.); gabrieledadalt@gmail.com (G.D.S.); garrastazugp@gmail.com (G.G.P.); adriana.pohlmann@ufrgs.br (A.R.P.); silvia.guterres@ufrgs.br (S.S.G.); 2 Food and Drug Department, University of Parma, Parco Area delle Scienze 27/a, 43124 Parma, Italy; stefania.pigana@studenti.unipr.it (S.P.); annalisa.bianchera@unipr.it (A.B.); laura.tiozzofasiolo@studenti.unipr.it (L.T.F.); 3 Programa de Pós-Graduação em Biociências, Universidade Federal de Ciências da Saúde de Porto Alegre, Porto Alegre RS 900500-170, Brazil; tanira@ufcspa.edu.br; 4 Department of Life Sciences and Biotechnology, University of Ferrara, Via Fossato di Mortara 17/19, 44121 Ferrara, Italy; clmgai@unife.it; 5 Programa de Pós-Graduação em Ciências Fisiológicas, Universidade Federal do Rio Grande, Rio Grande RS 96203-000, Brazil; magnomarques@aol.com; 6 Departamento de Química Orgânica, Instituto de Química, Universidade Federal do Rio Grande do Sul, Porto Alegre 91501-970, Brazil

**Keywords:** nasal permeability, nose-to-brain, simvastatin, nanocapsules, mucoadhesion, CNS disorders, chitosan

## Abstract

Drug delivery to the brain represents a challenge, especially in the therapy of central nervous system malignancies. Simvastatin (SVT), as with other statins, has shown potential anticancer properties that are difficult to exploit in the central nervous system (CNS). In the present work the physico–chemical, mucoadhesive, and permeability-enhancing properties of simvastatin-loaded poly-ε-caprolactone nanocapsules coated with chitosan for nose-to-brain administration were investigated. Lipid-core nanocapsules coated with chitosan (LNC_chit_) of different molecular weight (MW) were prepared by a novel one-pot technique, and characterized for particle size, surface charge, particle number density, morphology, drug encapsulation efficiency, interaction between surface nanocapsules with mucin, drug release, and permeability across two nasal mucosa models. Results show that all formulations presented adequate particle sizes (below 220 nm), positive surface charge, narrow droplet size distribution (PDI < 0.2), and high encapsulation efficiency. Nanocapsules presented controlled drug release and mucoadhesive properties that are dependent on the MW of the coating chitosan. The results of permeation across the RPMI 2650 human nasal cell line evidenced that LNC_chit_ increased the permeation of SVT. In particular, the amount of SVT that permeated after 4 hr for nanocapsules coated with low-MW chitosan, high-MW chitosan, and control SVT was 13.9 ± 0.8 μg, 9.2 ± 1.2 µg, and 1.4 ± 0.2 µg, respectively. These results were confirmed by SVT ex vivo permeation across rabbit nasal mucosa. This study highlighted the suitability of LNC_chit_ as a promising strategy for the administration of simvastatin for a nose-to-brain approach for the therapy of brain tumors.

## 1. Introduction

Statins are potent inhibitors of the hydroxymethyl glutaryl coenzyme A (HMG-CoA) reductase, and they are commonly administered for the treatment of cardiovascular disease [1]. However, in recent years, it has been suggested that the statin therapeutic indications might expand, due to their pleiotropic effects [2]. These non-cholesterol-related effects include their modulation of immune responses, the enhancement of endothelial function, the reduction of oxidative stress, and checking of inflammation processes [3,4]. The majority of pleiotropic effects are mediated by preventing the synthesis of isoprenoids, and the subsequent inhibition of small signaling proteins [5,6]. Among the diseases that could benefit from statin’s pleiotropic effects are multiple sclerosis, rheumatoid arthritis, systemic lupus erythematosus, chronic obstructive pulmonary disease, neurodegenerative disorders, bacterial infections, and cancer [3].

In the field of cancer therapy, statins showed pro-apoptotic effects against various tumor cell lines [7,8], and numerous studies have examined their potential chemopreventive action [9]. Due to the inhibition of the enzyme HMG-CoA reductase, statins decrease the levels of mevalonate, the precursor of dolichol, geranylpyrophosphate (GPP), and farnesyl-pyrophosphate (FPP). Dolichol enhances DNA synthesis and is associated with several proteins found in tumor cells [10]. GPP and FPP are post-translational modifications of intracellular proteins as the G-proteins Ras and Rho, which regulate the signal transduction of several membrane receptors, and which are critical for the transcription of genes involved in cell proliferation, differentiation, and apoptosis. Ras and Rho gene mutations are found in a variety of tumor cells [7,11]. Furthermore, statins show apoptotic effects in human glioblastoma cell lines that induce the depletion of geranylgeranylated proteins, which is important for the transition to cell cycle phases [12]. Statins have also shown to play a role in the prevention of tumor metastases by the inhibition of epithelial growth factor–induced tumor cell invasion [13]. Moreover, statins, inducing the inactivation of nuclear factor kB, reduce urokinase and matrix metalloproteinase-9 expression, which are pivotal for tumor metastatic processes [7,14]. 

In the case of glioma cell lines, simvastatin (SVT) showed a suppression of cell proliferation and the induction of apoptosis [15,16]. However, when evaluated in an in vivo orthotopic model of the glioblastoma multiform model, simvastatin did not show tumor-inhibitory effects [17]. As in this experiment, the major factor for the failure of chemotherapies against central nervous system (CNS) tumors has been attributed to limited brain-blood barrier (BBB) permeability [18]. In addition, after oral administration statins are extensively metabolized in the liver, their hydrophilic metabolites are prevented from crossing the BBB [19]. 

Some new strategies have been proposed to deal with such limitations in order to increase the distribution of drugs in the CNS, such as the use of nose-to-brain route [20,21] and the development of nanocarriers [22,23,24]. In recent years, nose-to-brain delivery has attracted much attention as a means of delivering drugs more efficiently to the CNS bypassing the BBB. This is because the nasal cavity is anatomically connected to the CNS via the olfactory system [25]. Moreover, it offers advantages such as non-invasiveness, the avoidance of hepatic first-pass metabolism, practicality and the convenience of administration [26]. However, due to the presence of the rapid mucociliary clearance mechanism, nasal delivery applications for brain delivery are hindered by the short residence time of conventional formulations. Moreover, the barrier of the nasal epithelium, nasal metabolism, and the limited volume of administration are limiting aspects for the development of nose-to-brain drug delivery systems [25,27]. 

To increase bioavailability after nasal delivery, polymeric nanocapsules have been investigated [28,29,30]. These nanocarriers are considered a type of reservoir drug delivery system [31], and can be obtained by the interfacial deposition of pre-formed polymers [32]. Their structure is characterized by an oil core surrounded by a polymeric shell stabilized by a surfactant system [31,33]. The lipid-core nanocapsules (LNCs), developed by our research group, are composed of a dispersion of sorbitan monostearate (solid lipid) and medium-chain triacylglycerols (liquid lipids) in the core, surrounded by poly(ε-caprolactone), an aliphatic polyester as a polymeric wall, and polysorbate 80 as a stabilizing surfactant [34]. The lipid core dispersion, i.e., sorbitan monostearate dispersed in oil, confers different properties to this system, such as controlling the drug release and increasing the encapsulation efficiency when compared to the core of the conventional nanocapsules containing only liquid lipids [34,35,36]. These nanocarriers showed efficient brain delivery of drugs as resveratrol [37] and curcumin [38] when administered orally and intraperitoneally, as well as the reduction of the side effects of the antipsychotic drug olanzapine [39]. Furthermore, they demonstrated improved the vitro and in vivo antitumor effectiveness of resveratrol, methotrexate, and acetyleugenol when compared to the free drugs [40,41,42]. 

Bender and co-authors [43], developed a two-step process to obtain modified LNC stabilized simultaneously with polysorbate 80, and lecithin and coated with chitosan. Chitosan is a cationic biopolymer obtained by the partial deacetylation of chitin under alkaline conditions [44]. Chitosan demonstrated several interesting properties for pharmaceutical application, such as biodegradability, biocompatibility, antibacterial activity, and the controlled release of drugs [45,46]. Furthermore, chitosan showed mucoadhesive and penetration-enhancing properties, which are particularly desirable for its application in drug nasal delivery [47,48]. These actions are mediated by the structural reorganization of the tight junctions of the nasal epithelium, increasing the paracellular transport of drugs [49]. 

In the present study, LNC stabilized with lecithin and coated with chitosan was obtained by an innovative one-pot technique. Moreover, the pharmaceutical properties of formulations with chitosans of different molecular weights (MW), intended for the nose-to-brain delivery of simvastatin, were evaluated.

## 2. Materials and Methods 

### 2.1. Materials

Poly (ε-caprolactone) (PCL) (MW = 14,000) and Span 60^®^ (sorbitan monostearate) were purchased from Sigma-Aldrich (Strasbourg, France). Caprylic/capric triglyceride was obtained from Delaware (Porto Alegre, Brazil), and simvastatin (SVT) was purchased from Pharma Nostra (Rio de Janeiro, Brazil). Chitosan low MW (21 kDa—viscosity 9 cP) and high MW (152 kDa—viscosity 114 cP) was provided by Primex (Chitoclear FG, deacetylation degree 95%, Siglufjordur, Iceland). Soybean lecithin (Lipoid S75) was kindly donated by Lipoid AG (Ludwigshafen, Germany). Minimum essential medium (MEM), fetal bovine serum (FBS), phosphate-buffered saline (PBS), and Hank’s Balanced Salt Solution (HBSS) were supplied by Gibco (Carlsbad, CA, USA). Transwell^®^ cell culture inserts (1.12 cm^2^ surface area, polyester, 0.4 µm pore size) were supplied by Corning Costar (Lowell, MA, USA). All of the other chemicals and solvents used were of analytical or pharmaceutical grade.

### 2.2. Preparation of the Lipid-Core Nanocapsules Coated using a One-Pot Technique

Chitosan-coated simvastatin-loaded lipid-core nanocapsules were prepared according to the method of interfacial deposition of pre-formed polymers already reported in literature [35]. An organic phase (25 mL of acetone) containing the polymer (PCL, 0.04 g), sorbitan monostearate (0.016 g), and caprylic/capric triglyceride (0.048 mL) was kept under magnetic stirring at 40 °C. After complete dissolution of the components, an ethanolic solution (4 mL) containing lecithin (0.025 g) was added into the organic phase, and finally, simvastatin (0.010 g) was added and completely dissolved. The aqueous phase (50 mL) contained 0.1% (*w*/*v*) chitosan, prepared as a dilution from a 0.5% (*w*/*v*) of chitosan solution in 1% (*v*/*v*) acetic acid. The organic phase was injected using a funnel into the aqueous phase under moderate magnetic stirring. The solvents were eliminated at 40 °C to a final volume of 10 mL, by the use of a rotary evaporator, Büchi^®^ R-114 (Flawill, Switzerland). The formulations obtained were named LNC_SVT-LMWchit_ when low-MW chitosan (viscosity 9 cP) was used, and LNC_SVT-HMWchit_ when high-MW chitosan (viscosity 114cP) was used. Blank nanocapsules (LNC_LMWchit_ and LNC_HMWchit_) were also prepared, omitting the simvastatin from the organic phase preparation.

### 2.3. Drug Content and Encapsulation Efficiency 

SVT quantification was carried out by high performance liquid chromatography with detection in the ultraviolet range (HPLC-UV), using a previously validated method [30]. The analysis was performed with a Shimadzu HPLC system (Kyoto, Japan) with detection at 238 nm and using a column Phenomenex Lichrosphere^®^ C18 (4.6 × 250 mm, 5 μm). The composition of the mobile phase was 65% acetonitrile and 35% sodium dihydrogen phosphate buffer (25 mM, pH 4.5), the flow rate was 1.0 mL·min^−1^, with an injection volume of 100 μL. Calibration curves (*n* = 3) were prepared to determine the linearity (*R* > 0.99) in the concentration range from 0.1 to 20 μg·mL^−1^. The drug content in the formulations was determined by diluting a precise volume of nanoparticle suspension (100 µL) in 10 mL of the mobile phase. The samples were then sonicated for 30 min and filtered through a 0.45 μm membrane (Millipore^®^, Billerica, MA, USA) before being assayed by HPLC-UV. Free simvastatin was determined in the ultrafiltrate after ultrafiltration–centrifugation (Ultrafree-MC, cut-off of 30 kDa, Millipore) at 2688× *g* (Scilogex D3024, Rocky Hill, CT, USA) for 15 min and quantification by HPLC-UV. Encapsulation efficiency (EE) as a percentage was calculated by the difference between the total and free, i.e., non-encapsulated, drug amount, divided by the total drug amount multiplied by 100. All analyses were performed for triplicate batches (*n* = 3).

### 2.4. Physicochemical Characterization

The nanoparticle formulations were characterized with multiple techniques, as described below. All analyses, with the exception of transmission electron microscopy (TEM) (*n* = 1), were performed for triplicate batches (*n* = 3).

#### 2.4.1. Laser Diffraction

Particle size and the size distribution were determined by laser diffraction (Mastersizer^®^ 2000, Malvern Instruments, Malvern, UK), with the aim of detecting the eventual presence of micrometric particles or aggregates. The sample was directly added to water in the wet dispersion accessory (Hydro 2000SM-AWM2002, Malvern Instruments) until an obscuration level of 2% was reached. The particle size was then expressed by using the volume-weighted mean diameter (D[4,3]), and the diameters calculated at the 10th, 50th, and 90th percentiles (*d*_0.1_, *d*_0.5_, and *d*_0.9_, respectively) of the cumulative size distribution curve, by volume (*v*) and by the number (*n*) of particles. The width of the distribution (*Span*) was determined according to Equation (1):

(1) $$Span=\frac{d_{0.9}-d_{0.1}}{d_{0.5}},$$

#### 2.4.2. Dynamic Light Scattering

The mean particle size (Z-average diameter) and the polydispersity index (PDI) of the nanocapsules were evaluated by dynamic light scattering (DLS) at 25 °C, using a Zetasizer^®^ Nano ZS (Malvern Instruments). After dilution of the samples (500×) in purified and filtered (0.45 µm) water, the correlogram was obtained by allowing the instrument software to determine the optimal time of acquisition, and the Z-average diameter and PDI were calculated by the method of Cumulants, with the same software.

#### 2.4.3. Nanoparticle Tracking Analysis

The nanoparticles tracking analysis (NTA) method was used to determine the mean diameter and the concentration of nanocapsules per volume, expressed as the particle number density (PND) (NanoSight LM10, Malvern Pananalytical, UK). The analysis was carried out by diluting the samples in ultrapure water (1000×) and introducing them into the instrument sample chamber cell by a syringe. The chamber is located on an optical microscope, where a laser diode (635 nm) illuminates the particles in suspension. The NTA 3.2 software tracks single particles, which are in Brownian motion, and it can relate this particle movement to a sphere equivalent hydrodynamic radius, as calculated using the Stokes–Einstein equation (Equation (2)). The samples were evaluated at room temperature for 60 s, with automatic detection. The results correspond to the arithmetic average of the calculated sizes of all particles analyzed:

(2) $$\bar{{\left(x,\;y\right)}^{2}}=\frac{4\;Tk_{B}}{3\pi \eta d_{h}}\;,$$

where $k_{B}$ is the Boltzmann constant, and $\bar{{\left(x,\;y\right)}^{2}}$ is the mean-squared displacement of a particle during time t at temperature *T*, in a medium of viscosity *η*, with a hydrodynamic diameter of $d_{h}$.

#### 2.4.4. pH and Zeta Potential

The pH values of the nanocapsules suspensions were determined using a calibrated potentiometer (DM-22 Digimed, São Paulo, Brazil) via direct measurements of the formulations at 25 °C. The zeta potential values were determined by electrophoretic mobility after the samples were diluted in 10 mM NaCl aqueous solution (500×), previously filtered (0.45 µm, Millipore^®^, Billerica, USA). The zeta potentials of the nanoparticles suspensions were also measured at different pH values, using the MPT-2 autotitrator for the Zetasizer^®^ Nano ZS (Malvern Instruments, Malvern, UK). The samples (10 mL) were placed in the titration cell and titrated over acid (0.1 M HCl) towards a basic (0.05 M NaOH) pH range in 1.0 pH unit intervals. This combination allowed for automated titration over a wide pH range, and thus, made it possible to determine the isoelectric point (IEP) of the nanoparticles.

#### 2.4.5. Morphology

TEM was used to evaluate the morphology of the formulations. TEM samples were diluted in ultrapure water (10×, *v*/*v*), and then deposited (10 µL) onto specimen grids (Formvar-Carbon support film, Electron Microscopy Sciences, Hatfield, PA, USA) and negatively stained with uranyl acetate solution (2% (*w*/*v*), Sigma-Aldrich, St.Louis, MO, USA). Analyses were performed using a transmission electron microscope (JEM 2200-FS, Jeol, Tokyo, Japan) operating at 80 kV. The images were processed with Digital Micrograph (Gatan Inc., Pleasanton, CA, USA) software.

### 2.5. In Vitro Evaluation of the Interaction between Nanocapsules and Mucin

The mucoadhesive properties of the formulations were assessed by using mucin from porcine stomach (Type II, Sigma-Aldrich, St. Louis, MO, USA), as previously described [29,50]. The mucin was dispersed in ultrapure water (0.5% (*w*/*v*)) by magnetic stirring for 3 h at room temperature. The suspension was centrifuged at 4000× *g* for 30 min. The supernatant was collected and lyophilized. Then, mucin solutions were prepared in a simulated nasal electrolytic solution (SNES) [51] at predetermined weight ratios f, determined as:

(3) $$f=\frac{W_{mucin}}{W_{mucin}+\;WNC}\;,$$

where *W*_mucin_ is the mucin mass and *WNC* is the LNC_chit_ nanocapsule mass.

The Z-average diameter and PDI before and after contact with mucin were measured by DLS as described above, after the dilution (500×) of the samples in mucin solutions. The mucoadhesive index values (MI) were determined as:

(4) $$MI=\frac{d}{d_{0}},$$

where *d* and *d*₀ are the diameters of the LNC_chit_ nanocapsules before and after their interaction with mucin, respectively.

Furthermore, changes in the zeta potential were measured after the nanocapsules were diluted (500×) in mucin solutions containing 10 mM NaCl.

### 2.6. In Vitro Release Study

The in vitro release profiles of SVT from the formulations were determined by using the dialysis bag method. Briefly, 1 mL of each sample was placed in a dialysis bag (14 kDa molecular weight cut-off, Sigma-Aldrich, St. Louis, MO, USA) and suspended into 100 mL of SNES containing 0.5% of polysorbate 80, to improve SVT solubility and to reach the sink conditions. A free drug solution was placed in the dialysis bag in the control experiment (SVT_solution_, simvastatin dissolved in 1% ethanol and 0.5% polysorbate 80). The dialysis bags were maintained in the medium under stirring, and maintained in a temperature-constant water bath (37 °C). One milliliter of release medium was collected at predetermined time intervals (from 0.16 to 8 h) and filtered (0.45 µm, Millipore^®^). The volume was replaced by adding one milliliter of fresh release medium pre-heated at 37 °C. The samples were analyzed by HPLC–UV, and the cumulative drug release was determined.

### 2.7. Transport Studies across an In Vitro Nasal Epithelial Cell Model

RPMI 2650 human nasal cells (human nasal septum tumor, ECACC, Salisbury, UK) were cultured in MEM media supplemented with 10% (*v*/*v*) fetal bovine serum (FBS). Cells were grown at 37 °C in an atmosphere of 95% air/5% CO_2_. Transwell^®^ cell culture inserts were used to establish an air–liquid interface (ALI) nasal model, as previously reported [52]. Briefly, 200 uL of cell suspensions (2.5 × 10^6^ cell/mL) were seeded onto Transwell^®^ and after 24 h, the media from apical compartment was removed, resulting in an ALI culture configuration. After 14 days from seeding, the Transwell^®^ was removed and transferred to a 12-well plate containing 1.5 mL of pre-warmed HBSS. Then, 200 µL of LNC_SVT-LMWchit_, LNC_SVT-HMWchit_, and SVT_suspension_ were added to the upper compartment, and samples of 200 µL were collected from the baso-lateral chamber at pre-determined time points (1, 2, 3 and 4 h) and replaced with the same volume of fresh HBSS buffer. The samples were quantified for simvastatin content using HPLC–UV (*n* = 4). TEER measurements were performed with a Millicell-ers^®^ (Millipore) at the beginning and at the end of experiment, in order to confirm that the integrity of the cell layer was maintained.

### 2.8. Ex Vivo Transport Experiments across Rabbit Nasal Mucosa

The transport of simvastatin across rabbit nasal tissue was evaluated by using Franz-type vertical diffusion cells with a receptor volume of 4.5 mL and a diffusional area of 0.58 cm^2^. On the day of the experiment, nasal mucosae were freshly excised from rabbits obtained from a local slaughterhouse (Pola, Finale Emilia, Italy), and cleaned to remove the adhering submucosal tissue [53]. The rabbit nasal mucosa were placed between the donor and the receptor compartments of the diffusion cells. Then, in order to check the mucosa integrity, the donor compartment was filled with medium to confirm that no liquid leaked into the cell receptor compartment. If the nasal mucosa passed this test, the donor compartment received 200 µL of one of the three tested preparations, i.e., LNC_SVT-LMWchit_, LNC_SVT-HMWchit_, or SVT_suspension_, equivalent to 200 µg of SVT. The Franz cells were maintained at 37 °C under mild magnetic stirring. At predetermined time intervals, 500 µL of the receptor medium, SNES containing 0.5% w/v of polysorbate 80, was withdrawn, and the receptor compartment was refilled with an equivalent volume of fresh medium. All samples were analyzed by HPLC-UV. In order to evaluate the retention of SVT in the nasal tissue, mucosa samples were placed in a volumetric flask (10 mL) containing a solvent of extraction (acetonitrile), and subjected to vortexing (2 min) and sonication (15 min). The solvent of extraction was then filtered (0.45 µm, Millipore^®^) and simvastatin was quantified using HPLC–UV. The experiments were conducted in triplicate for each formulation.

### 2.9. Preliminary Nasal Toxicity Studies

Nasal toxicity studies were performed by using rabbit nasal mucosa in a similar way to the ex vivo permeation study mentioned above, in order to evaluate any damage to the mucosa. Each mucosa piece of uniform thickness was treated with LNC_SVT-LMWchit_, LNC_SVT-HMWchit_, SVT, or PBS pH 6.4 (negative control) by placing 200 µL in the donor compartments of the Franz diffusion cells. The acceptor contained 4.5 mL of SNES containing 0.5% (*w*/*v*) of polysorbate 80. After 4 h, for each condition, the nasal mucosa was washed with PBS, fixed in 10% (*v*/*v*) buffered formalin for 6 h, and embedded in histological paraffin. A rotatory microtome was used to perform transverse cuts to obtain sections (5 µm) that were stained with hematoxylin–eosin. Images of mucosa samples were observed by using an Olympus BX51 optical microscope with an attached DP72 camera (Olympus, Tokyo, Japan) [54].

### 2.10. Statistical Analysis

Data are presented as the mean ± standard deviation (SD) of the analysis of at least triplicate batches (*n* = 3). Statistical analysis was performed using the Student’s t-test for two groups, or one-way analysis of variance (ANOVA) followed by Tukey’s test for multiple groups, using GraphPad Prism Software 5.0 (GraphPad Software, Inc., San Diego, CA, USA). Differences were considered significant at *p* < 0.05.

## 3. Results

SVT-nanocapsules were prepared by using the interfacial deposition of pre-formed polymer method, and coated with chitosan with a one-pot technique. The surfaces of the NPs were coated with two different chitosan grades, characterized by different molecular weights, and hence different viscosities, in order to evaluate the effect of chitosan molecular weight on the mucoadhesive properties and permeability enhancement of the nanocapsules obtained.

### 3.1. Characterization of Nanocapsules

**Figure 1:**
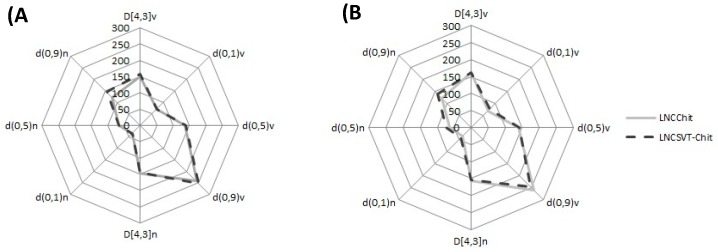
Radar chart presenting the volume-weighted mean diameters (D[4,3]) and the diameters at percentiles 10, 50, and 90 under the size distribution curves by volume and by the number of particles. Chitosan-coated simvastatin-loaded lipid-core nanocapsules developed with (**A**) low-MW chitosan and (**B**) high-MW chitosan.

SVT nanocapsules appeared macroscopically as an opalescent white homogeneous dispersion. The total SVT content in the formulations was found to be 0.94 ± 0.04 mg·mL^−1^ for LNC_SVT-LMWchit_ and 0.96 ± 0.02 mg·mL^−1^ for LNC_SVT-HMWchit_. Regarding the encapsulation efficiency (EE), SVT was not detected in the ultrafiltrate for both formulations, indicating almost complete encapsulation. The high EE achieved is probably linked to the high SVT distribution coefficient (log D) of 4.72, which confirms its great affinity for the lipophilic phase, and its concentration in the core of the nanocapsules [55]. Laser diffraction (LD) analysis showed D[4,3] of 150 ± 7 nm (LNC_LMWchit_), 157 ± 6 nm (LNC_HMWchit_), 163 ± 2 nm (LNC_SVT-LMWchit_), and 161 ± 3 nm (LNC_SVT-HMWchit_), with Span values of 1.3 ± 0.1, 1.3 ± 0.1, 1.4 ± 0.03, and 1.2 ± 0.2, respectively. According to the results obtained with this technique, there were no significant differences in terms of the particle diameter and polydispersity, between the formulations with and without SVT (*p* > 0.05). The shapes of the curves in the radar chart presented in Figure 1 are fingerprints of the formulations, which demonstrate the narrow size distributions and confirm their low polydispersities [56]. All of the formulations showed similar behaviors and they had *d*_0.9_ calculated both for the cumulative distribution by number, and by a volume lower than 300 nm (Figure 1).

The mean particle size was further confirmed by DLS and NTA analysis, as shown in Table 1. The DLS analysis showed a narrow particle size distribution for all of the formulations (PDI < 0.2). In particular, the encapsulation of SVT did not affect the particle size of the nanocapsules coated with low-MW chitosan. In fact, the diameters of LNC_LMWchit_ and LNC_SVT-LMWchit_ were not significantly different (*p* > 0.05).

**Figure 2:**
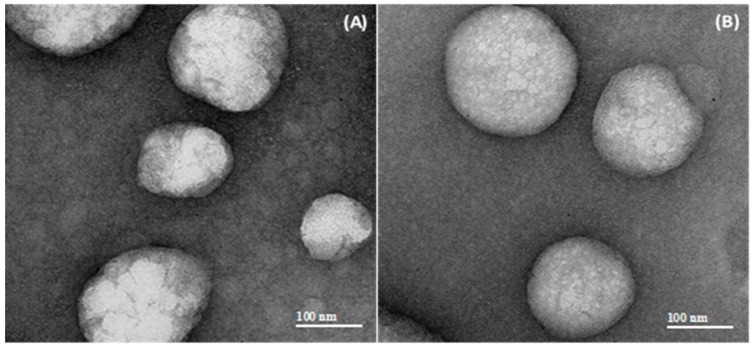
Transmission electron microscopy (TEM) micrographs (magnification 40,000×) of chitosan-coated lipid core nanocapsules: (**A**) LNC_SVT-LMWchit_ and (**B**) LNC_SVT-HMWchit_.

On the other hand, the presence of SVT led to a significant size increase (*p* ≤ 0.05) in the case of LNC_SVT-HMWchit_ (210 ± 10 nm) when compared with the blank nanocapsules (LNC_HMWchit_, 188 ± 7 nm) according to the NTA analysis, but not according to the DLS data. This difference may be explained by the formation of a small number of particle agglomerates, compared to the formulation prepared without the drug. In addition, these agglomerates appeared to be detected more efficiently by the NTA technique compared to DLS. This is supported by the particle number density (PND) also measured by NTA, which evidenced, upon SVT encapsulation, almost a halving of the particle number density (PND), which was 1.1 ± 0.4 × 10^12^ particles/mL for LNC_HMWchit_ and 6.6 ± 0.2 × 10^11^ particles/mL for LNC_SVT-HMWchit_ (Table 1). Furthermore, in general, the nanocapsules coated with HMW chitosan showed a slightly higher mean diameter than those coated with LMW chitosan (*p* > 0.05), probably as a consequence of the higher molecular weight and higher viscosity of the chitosan in the water phase. In general, it has been reported that the higher the viscosity of the dispersion medium, the higher the mean diameter of the nanoparticles produced [57]. The zeta potentials of LNC_LMWchit_ and LNC_HMWchit_ were similar to those determined for the SVT-loaded nanocapsules. Therefore, the encapsulation of SVT did not significantly affect this parameter (*p* > 0.05). The pH value at which the nanoparticles do not exhibit any net charge is termed the isoelectric point (IEP). LNC_SVT-LMWchit_ and LNC_SVT-HMWchit_ showed an IEP at pH value 7.1 ± 0.2 and 7.2 ± 0.5, respectively. The positive zeta potential obtained for all the formulations prepared, and an IEP near the IEP of chitosan (IEP = 6.8) [58] is a good indicator that the chitosan was present at the external interface of the nanocapsules. LNC_SVT-LMWchit_ and LNC_SVT-HMWchit_ were further characterized in terms of their morphologies (Figure 2).

Concerning the nanocapsule morphology, TEM images clearly displayed spherical-shaped particles, with a core with low electron density, as expected for lipid nanocapsules. Also, from the results of the particle diameters, the images were in good agreement with the mean nanocapsule sizes determined with DLS, NTA, and LD.

### 3.2. Mucoadhesion Studies

**Figure 3:**
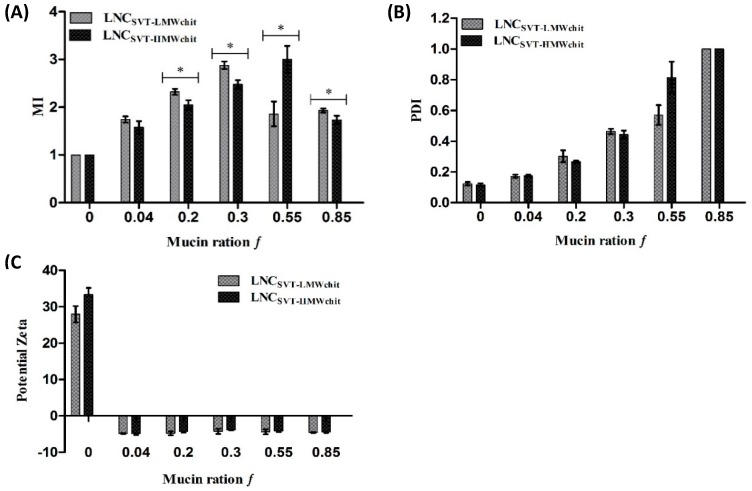
(**A**) Mucoadhesive index (MI) values, (**B**) PDI and (**C**) zeta potentials measured for various mixtures of mucin and nanocapsules. Values of the two formulations were obtained before (*f* = 0) and after incubation with different mucin weight ratios *f*.

To investigate the interaction between the formulations and mucin, a mucoadhesion index (MI), the PDI, and the zeta potentials of mixtures between the nanocapsules and mucin were determined for different mucin weight ratios f (Figure 3). For LNC_SVT-LMWchit_ in the range of *f* values up to 0.3, particle sizes and MI values increased to a maximum of 474 ± 6 nm and 2.9 ± 0.1 (*f* = 0.3), respectively. Similarly, LNC_SVT-HMWchit_ particle size and MI increased in the f range from 0 to 0.55, reaching a maximum of 557 ± 30 nm, 3.0 ± 0.3 (*f* = 0.55), respectively (Figure 3A). Despite the increase of the fraction by weight of mucin above f values of 0.3, for LNC_SVT-LMWchit_, particle size and MI decreased to 318 ± 5 nm and 1.9 ± 0.1 for *f* values of 0.85. It has been hypothesized here that while at values of f of around 0.3, nanocapsules are able to form large agglomerates with mucin chains entangled and linked with more nanoparticles, above this value, the mucin is in such a large excess that almost all single particles are enrobed with mucins, leading to a decrease in the overall mean particle size. In fact, the MI values for f = 0.55 and 0.85 did not change significantly. On the other hand, for LNC_SVT-HMWchit_ this decrease in the MI occurred only for *f* values above 0.55, with particle size and MI decreasing to 321 ± 7 nm, 1.7 ± 0.1 (*f* = 0.85). Therefore, the MI maximum was observed at lower f values (0.3) for LNC_SVT-LMWchit_, and higher f values (0.55) for LNC_SVT-HMWchit_, indicating a higher capacity to interact with larger quantities of mucin for nanoparticles coated with high MW chitosan, macroscopically translating to more efficient mucoadhesion.

The PDI before nanocapsule interaction with mucin was 0.12 ± 0.01 for LNC_SVT-LMWchit_ and 0.11 ± 0.01 for LNC_SVT-HMWchit_. After mixing the nanocapsules with mucin, PDI progressively increased for both formulations, up to 1 (*f* = 0.85), indicating the formation of agglomerates and the switch to highly polydispersed particle size distributions (Figure 3B). The zeta potentials of the formulations that were initially positive due to the chitosan coating immediately after interaction with even the smallest amount of mucin (*f* = 0.04), became negative for both formulations (−4.8 ± 0.1 mV for LNC_SVT-LMWchit_ and −4.8 ± 0.7 mV for LNC_SVT-HMWchit_) and remained roughly constant for both formulations, even with increasing f values (Figure 3C).

### 3.3. In Vitro Drug Release

**Figure 4:**
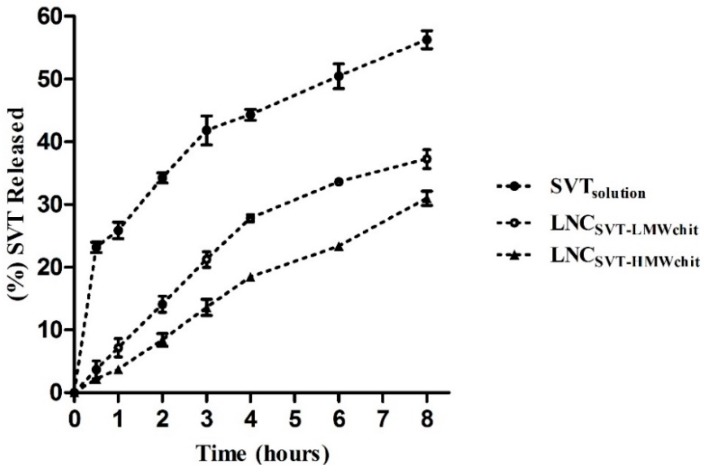
In vitro drug release profile from LNC_SVT-LMWchit_, LNC_SVT-HMWchit_ and from the control (SVT solution), using the dialysis bag method at 37 °C (*n* = 3, ± SD).

The in vitro release profiles of the simvastatin-loaded chitosan coated lipid core nanocapsules were performed over 8 h (Figure 4). A solution of simvastatin was used as a control. It can be observed that 56.3 ± 2.5% of SVT diffused out from the dialysis bag when using the control simvastatin solution, within 8 h. On the other hand, the percentage of drug released from LNC_SVT-LMWchit_ and LNC_SVT-HMWchit_ after 8 h were 37.3 ± 1.5% and 31.0 ± 1.1%, respectively. These results show that nanocapsules provided a controlled release of simvastatin, and that the different types of chitosan affected the drug release rate. Indeed, the release of SVT from LNC_SVT-HMWchit_ was slower in comparison to the LNC_SVT-LMWchit_.

### 3.4. Transport Studies in a Nasal Cell Model

**Figure 5:**
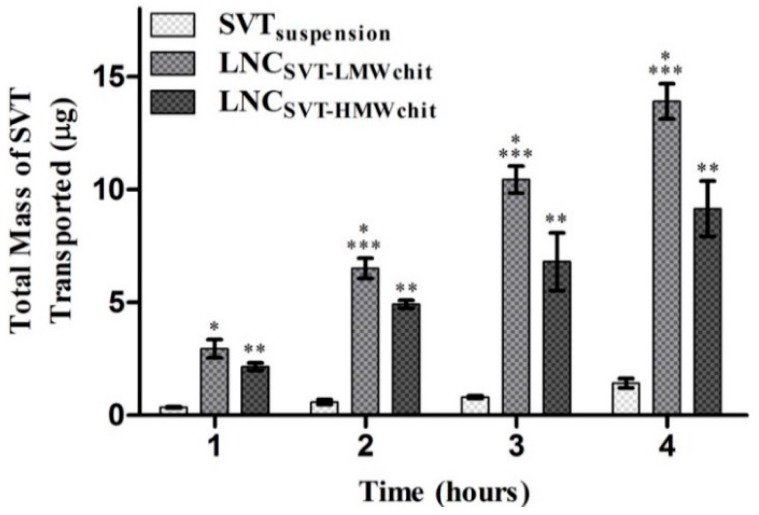
Amount (µg) of SVT transported across RPMI 2650 cells grown under air–liquid interface conditions (*n* = 4, ± SD). Significant difference (*p* < 0.05) is expressed considering the following comparisons: * SVT versus LNC_SVT-LMWchit_, ** SVT versus LNC_SVT-HMWchit_, *** LNC_SVT-LMWchit_ versus LNC_SVT-HMWchit_.

To confirm the potential permeability enhancement effects of the nanocapsules, the total amount of SVT transported across nasal mucosa model was determined by using RPMI2650 human nasal epithelial cells [52]. Figure 5 shows the amount of SVT transported when using the formulations, compared to a suspension of the raw material, over 4 h. After 1 h, the formulations already showed increased degrees of permeation compared to the control. However, no significant differences (*p* > 0.05) were observed at 1 h for the SVT permeation between LNC_SVT-LMWchit_ and LNC_SVT-HMWchit_. After the first hour however, each single time point showed an amount of SVT transported to be statistically different between the formulations (*p* < 0.05), confirming the greater permeation of LNC_SVT-LMWchit_.

### 3.5. Ex Vivo Transport Experiments across Rabbit Nasal Mucosa

**Figure 6:**
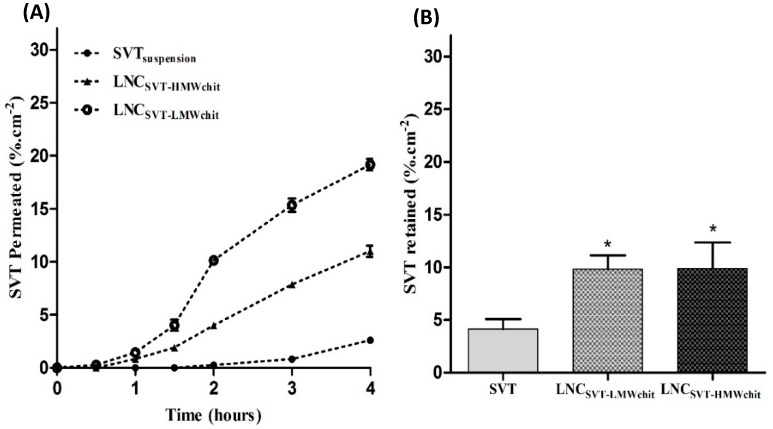
(**A**) Ex vivo SVT permeation across rabbit nasal mucosa up to 4 h in simulated nasal electrolytic solution (SNES) containing 0.5% of polysorbate 80 at 37 °C (*n* = 3, ± SD). (**B**) Percentage of SVT retained in nasal mucosa after 4 h of the permeation test in Franz-type diffusion cells (*n* = 3, ± SD). Asterisk (*) indicates significant differences between SVT versus LNC_SVT-LMWchit_ and LNC_SVT-HMWchit_.

Figure 6A shows the SVT permeation across the excised rabbit nasal mucosa using Franz type vertical diffusion cells. The results of the ex vivo permeation studies on the rabbit nasal mucosa evidenced that the formulations significantly increased the permeation of SVT compared to the control. LNC_SVT-LMWchit_ showed the highest percentage of SVT permeation after 4 h (19.2 ± 0.5%·cm^−2^), followed by LNC_SVT-HMWchit_ (11.0 ± 0.5%·cm^−2^), while the control (SVT suspension) showed an extremely low degree of permeation (2.6 ± 0.2%·cm^−2^). The results indicate that SVT permeation from LNC_SVT-LMWchit_ was 1.74 and 7.4 times greater than LNC_SVT-HMWchit_ and the SVT control, respectively.

The results of SVT retained in the mucosa tissue after 4 h experimental time are shown in Figure 6B. It can be seen that there was better retention of both formulations with respect to the control. The SVT fraction found in the tissue for the control was 4.2 ± 0.9%·cm^−2^, while in the case of LNC_SVT-LMWchit_ and LNC_SVT-HMWchit_, the fraction of simvastatin found in the tissue was more than doubled, i.e., 9.8 ± 1.3 and 9.9 ± 2.5%·cm^−2^, respectively. In this case, the difference between the amount of retained SVT from LNC_SVT-LMWchit_ and LNC_SVT-HMWchit_ was not statistically significant.

### 3.6. Preliminary Nasal Toxicity Studies

**Figure 7:**
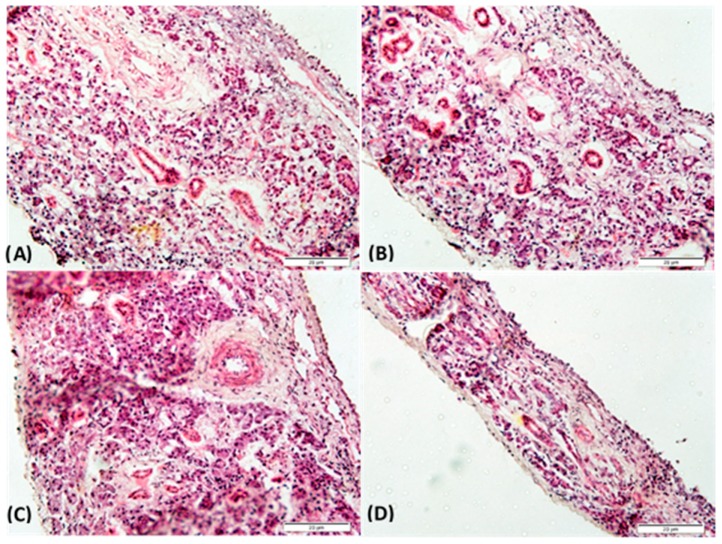
Histopathological sections of rabbit nasal mucosa after 4 h of permeation test in Franz-type diffusion cells treated with (**A**) PBS pH 6.4 (negative control), (**B**) SVT, (**C**) LNC_SVT-LMWchit_, and (**D**) LNC_SVT-HMWchit_. Sections are stained with hematoxylin and eosin.

Nasal mucosa histopathology studies were performed to assess the integrity of the rabbit mucosa after 4 h of the permeation test. As shown in Figure 7, the mucosa treated with PBS pH 6.4 (negative control), SVT, LNC_SVT-LMWchit_, and LNC_SVT-HMWchit_ did not show any evident structural damage. The results from histological examinations indicated that the LNC nanocapsules did not cause any irritation or toxicity, and they can be considered to be biocompatible for nasal administration.

## 4. Discussion

Over the past couple of decades, pharmaceutical nanotechnology has received considerable attention and demonstrated significant potential for the development of innovative medicinal products, both for the therapy and the diagnosis of severe diseases [59]. In this work, we report a new preparation method of lipid-core PCL nanocapsules, optimizing a two-step process (self-assembly step followed by a coating step) into a one-pot technique to develop chitosan-coated nanoparticles. Moreover, we compared the pharmaceutically relevant properties in terms of physicochemical characterization, mucoadhesive properties, drug release, and permeability studies of LNC_SVT-LMWchit_ and LNC_SVT-HMWchit_ coated by chitosan with low MW and high MW, in view of a nose-to-brain administration.

Polymeric nanocapsules containing poly(ɛ-caprolactone), capric/caprylic triglyceride, and sorbitan monostearate, and coated with chitosan, were developed. This formulation has the advantage of efficiently encapsulating lipophilic drugs such as simvastatin [34,35]. The production of positively-coated lipid-core nanocapsules in one step, avoiding the use of non-ionic surfactants in the aqueous phase, is the main novelty of this approach. Previously, we developed mucoadhesive amphiphilic nanocapsules based on a blend of PCL and poly(methyl methacrylate-b-2-(dimethylamino)ethyl methacrylate) for the nose-to-brain delivery of olanzapine. Nevertheless, the self-assembly of the nanocapsules by using this block copolymer (positive surface) avoids the use of sorbitan monostearate in the formulation. Indeed, the cores of those nanocapsules are composed exclusively of medium-chain triglycerides [29]. Sorbitan monostearate dispersed in core of the lipid-core nanocapsules has been demonstrated to provide an addition diffusional barrier to control the drug release, and an increase of the rigidity of the nanocapsules polymer wall [34,36]. Furthermore, a comparative study conducted with polymeric nanocapsules demonstrated that the presence of sorbitan monostearate instead of sorbitan monooleate in the core improved the anti-apoptotic and antioxidant effects of melatonin during bovine embryo development [60]. In the present development, it was possible to obtain in a one-step process, lipid-core nanocapsules incorporating sorbitan monostearate in the oily core, and decorated on the surface with chitosan, providing mucoadhesion and penetration-enhancing properties that appear to be fundamental for nose-to-brain delivery [21].

The combination between the composition and the interfacial deposition of the pre-formed polymer method produced nanocapsules with adequate particle sizes and narrow size distributions, as was confirmed by complementary techniques such as laser diffraction, DLS, and NTA. Besides that, coating the nanocapsules with chitosan with different molecular weights (21 kDa and 152 kDa) influenced their pharmaceutical properties. For example, chitosan molecular weight and viscosity in water affected the sizes of the nanoparticles. In fact, particle size could be reduced by using lower molecular weight chitosan [61,62]. Actually, for LNC_SVT-HMWchit_, the higher mean particle hydrodynamic diameter could be attributed to the lengths of the chains of chitosan present on the particle surface, possibly expanding the water hydration shell of the nanoparticle. In addition, low-MW chitosan has been reported to have a higher aqueous solubility, and this, together with shorter polymer chains, contributes to forming smaller particles compared to high-MW chitosan [63]. The positive zeta potential obtained for all formulations is evidence of the polysaccharide coating. Furthermore, using different chitosan types did not cause significant changes in the zeta potentials of both formulations, probably due of a similar degree of deacetylation (95%) [64]. Mucoadhesive polymers such as chitosan represent a significant strategy for overcoming the nasal drug delivery limits of low membrane permeability, short residence time, and mucociliary clearance. The presence of polysaccharides on the nanocapsule surface is expected to prolong the permanence of the formulation in the nasal cavity, open the tight junctions between the nasal epithelial cells, and promote drug permeation through the biological barriers, granting access to the CNS [65,66,67]. For these reasons, the evaluation of the ability of nanocapsules to interact with mucin is of great interest for nasal administration. Mucous membranes internally delimit the body cavities (stomach, esophagus, cornea, oral cavity, reproductive and respiratory tracts), and are characterized by a superficial mucus layer with protective and lubricating functions [68]. The mucus is composed of water (approximately 95%), lipids, inorganic salts, and mucin, a glycoprotein composed of *N*-acetylgalactosamine, *N*-acetylglucosamine, fucose, galactose, and sialic acid, which is responsible for the adhesive properties and the viscosity of the mucus [66]. Lipophilic drugs show an affinity for mucus glycoproteins, reducing their adsorption and the bioavailability. This issue, together with the drugs’ poor aqueous solubilities, are limiting factors for the nose-to-brain delivery of lipophilic drugs. To improve the transport of SVT through this barrier, we used nanoparticles as a drug delivery system [69].

Our results showed how, for both formulations, particle size and MI increased with the increase in the mucin weight ratio, to a critical point. A previous study [50] has led to the conclusion that the nanoparticles in mucin solution form agglomerates in which the nanoparticles are the points of contact between negatively charged mucin chains. Beyond the critical value of the mucin ratio, the repulsive forces among the mucin chains break these agglomerates. Our results support this theory; nevertheless it has to be stressed that the decrease of MI observed for high mucin weight ratio does not indicate a decrease in the mucoadhesive capacity of the formulations [50]. However, the results indicate how in mucin excess, smaller aggregates are formed in order to minimize the repulsive forces between the chains of mucin, and to maximize the points of contact between the positive charges of chitosan and the negative charges of the mucin chains. Moreover, it was noticed that the critical point of the formulations was different for the nanocapsules coated with chitosan with different characteristics (LNC_SVT-LMWchit_ *f* = 0.3 and for LNC_SVT-HMWchit_ *f* = 0.55). This difference could be attributed to the different chain lengths of the two chitosan batches [63]. Indeed, in a previous study conducted with a different method, Menchicchi and co-authors proposed that mucin interacts mostly with high molecular weight chitosan [70]. On the other hand, the results presented in this paper, compared to similarly uncoated LNC, demonstrate a large increase in the mucoadhesive capacity, due to the presence of chitosan. The uncoated LNC showed values of MI = 1.4, PDI = 0.51, and a zeta potential of −9.5 mV after contact with the maximum concentration of mucin (0.5% *w*/*v*) [71]. The PDI increase with the increase in the *f* mucin ratio corroborates the above-mentioned explanation. In fact, PDI results indicated that the heterogeneity of the distribution of the particle size in solution increased along with the mucin weight ratio, because of the formation of aggregates of different sizes. The oligosaccharide chains of mucin glycoproteins presenting terminal sialic acid residues confer a negative charge to the molecule; therefore, the variation of the particle zeta potential from positive to negative values demonstrates how the mucin enrobes the nanocapsules interacting with their surface layer of chitosan. This suggests that mucoadhesion mechanism is driven by the electrostatic interaction of the positively charged amine groups of d-glucosamine molecules of chitosan with the negatively charged sialic acid residues of mucin [72].

The SVT-loaded lipid core nanocapsules formulation was able to control drug release, due to the two diffusional barriers: the PCL polymer wall and the lipid dispersion present in the nanocapsule core. The nanoencapsulation reduced the diffusion rate of SVT across a dialysis membrane, confirming data that is frequently described in the literature as a property of polymeric nanoparticles [33]. Regarding the comparison between the two formulations, LNC_SVT-HMWchit_ afforded better control of drug release. In order to explain the different release profiles, we evaluated the differences in terms of their physicochemical properties. Firstly, this can be explained by the higher viscosity of the chitosan used to develop the LNC_SVT-HMWchit_. Previous studies demonstrated that the viscosity of the chitosan is an important factor for modulating the release control [73]. Moreover, nanoparticle size can influence in the release profile, which increases with decreasing particle size. Indeed, LNC_SVT-LMWchit_ had a lower mean particle diameter than LNC_SVT-HMWchit_, which could contribute to explaining their different release profiles, because of the greater available surface area [74,75].

The permeation across the RPMI 2650 human nasal cell line showed that the SVT transported across the nasal cell model by LNC_SVT-LMWchit_ and LNC_SVT-HMWchit_ can enhance drug transport across a cell pseudo-monolayer that also secretes mucin on its apical surface. The increase of SVT permeation across the human nasal cell layer can be explained by different mechanisms. In literature, chitosan is associated with an opening of tight junctions that increase cell layer permeability. However, this mechanism generally favors the permeation of water-soluble drugs, and it has been evidenced that it is slightly less efficient for chitosan bound to nanoparticles, compared to simple polysaccharide solutions [76]. More recently, it has been demonstrated that the biodegradation of nanocapsules by enzymes in the mucus barrier or intracellularly could be pivotal in enhancing the transcellular transport of lipophilic drugs [77]. The results of the ex vivo permeation studies on rabbit nasal mucosa confirmed and further added evidence for the fact that the nanoencapsulation of SVT significantly increased its permeation across excised nasal mucosa. In this case, the higher permeation evidenced for LNC_SVT-LMWchit_ could be explained by a combination of factors. The smaller particle sizes of nanocapsules coated with low molecular weight chitosan could have facilitated its absorption into the mucosal tissue as reported before [78]. Moreover, an important factor is the bioadhesion of the nanostructures, as previously explained, given by the particles shell of chitosan, which interacts with electrostatic forces at the anionic sites of the nasal mucus [65]. However, from the mucoadhesion data, a better interaction with mucus could be expected from nanocapsules coated with the high molecular weight chitosan. However, the slower release kinetics evidenced for these particles have probably contributed to limiting the amount of drug permeated, especially considering that drug accumulation into the tissue is superimposable for the two nanocapsule formulations.

Previous findings [79,80], agree that chitosan-coated nanoparticles increase drug permeation across the nasal mucosa, compared to the free drug control. This controlled SVT permeation, together with the mucoadhesion effects, may be an important strategy for prolonging the effects of the drug when it is administered via the nasal route.

## 5. Conclusions

In the present investigation, the coating process of lipid-core nanocapsules, using a one-pot technique approach, was presented. Furthermore, two chitosan-coated simvastatin-loaded lipid core nanocapsules suitable for nasal administration were successfully developed. Both the nanoparticles produced with different types of chitosan were designed to have adequate physicochemical and mucoadhesive properties for a potential nose-to-brain application. The formulations prepared combine a controlled release of simvastatin, and mucoadhesion properties that are able to increase drug permeation across the nasal mucosa, as demonstrated by using two different models of nasal epithelium. In summary, simvastatin-loaded chitosan-coated lipid core nanocapsules seem to be a promising mucoadhesive system for the nose-to-brain delivery of poorly soluble anticancer drugs. Follow up studies will focus on the investigation of the potential of these nanoparticles for the treatment of brain tumors, involving studies with glioma cells, and an orthotopic intracranial tumor model in mice.

## Figures and Tables

**Table 1:** Physicochemical characterization of the nanocapsules (*n* = 3, Mean ± Standard Deviation).

Header	DLS		NTA		Zeta Potential (mV)	pH
	*Z*-Average (nm)	PDI	Mean (nm)	PND (Particles/mL)		
LNC_LMWchit_	166 ± 5	0.13 ± 0.02	174 ± 5	1.3 ± 0.3 × 10^12^	25.4 ± 4.1	4.1 ± 0.01
LNC_SVT-LMWchit_	168 ± 5	0.12 ± 0.04	166 ± 7	1.2 ± 0.6 × 10^12^	28.95 ± 2.1	4.1 ± 0.02
LNC_HMWchit_	179 ± 14	0.13 ± 0.02	188 ± 7	1.1 ± 0.4 × 10^12^	33.6 ± 3.9	4.1 ± 0.03
LNC_SVT-HMWchit_	185 ± 7	0.16 ± 0.03	210 ± 10	6.6 ± 0.2 × 10^11^	33.8 ± 5.5	4.4 ± 0.04
